# Placenta-derived exosomes exacerbate beta cell dysfunction in gestational diabetes mellitus through delivery of miR-320b

**DOI:** 10.3389/fendo.2023.1282075

**Published:** 2024-01-08

**Authors:** Yanmei Wang, Yue Yuan, Shanmei Shen, Zhijuan Ge, Dalong Zhu, Yan Bi

**Affiliations:** ^1^ Department of Endocrinology, Endocrine and Metabolic Disease Medical Center, Nanjing Drum Tower Hospital, Affiliated Hospital of Medical School, Nanjing University, Nanjing, China; ^2^ Branch of National Clinical Research Centre for Metabolic Diseases, Nanjing, China

**Keywords:** gestational diabetes mellitus, placenta-derived exosome, β-cell dysfunction, microRNA, miR-320b

## Abstract

Recent studies have shown placenta-derived exosome (pdE) acts as an important mediator of organ-to-organ interplay regulating maternal metabolic alterations, however, the function and mechanisms of placental exosomes on pancreatic β-cell maladaptation in gestational diabetes mellitus (GDM) remain unclear. The purpose of this investigation was to ascertain how placental exosomes affected the β-cell dysfunction associated with the onset of GDM. Exosomes were isolated from chorionic villi explants of pregnant mice and humans with normal glucose tolerance (NGT) and GDM. The effects of pdE from GDM on glucose tolerance *in vivo* and islets function *in vitro* were determined. Isolated islets from mice fed on the chow diet displayed an increase in apoptosis and observed their glucose-stimulated insulin secretion (GSIS) greatly diminished by PdE from GDM mice. Mice that accepted PdE from mice with GDM possessed glucose intolerance.Based on miRNA microarray assay and bioinformatics analysis from human placental exosomes, we identified miR-320b selectively enriched in PdE secreted in GDM compared with NGT. Importantly, the level of placental miR-320b was positively correlated with the 1h-glucose and 2-h glucose of a 75 g oral glucose tolerance test (OGTT) during human pregnancies. Furthermore, miR-320 overexpression attributed to impaired insulin secretion and increased apoptosis in MIN6 cells and islets obtained from mice with normal insulin sensitivity. This study firstly proposed that altered miRNAs in pdE contribute to defective adaptation of β cells during pregnancy, which expands the knowledge of GDM pathogenesis. Exosomes from the placenta may be an emerging therapeutic target for GDM.

## Introduction

Gestational diabetes mellitus (GDM) is defined as glucose intolerance with a variety of severity that is initially noticed during pregnancy (1). GDM is associated with perinatal adverse outcomes, an increased risk of cardiovascular disease and type 2 diabetes in the mother and her offspring (2–5). Maternal metabolism is programmed through the development of maternal relative insulin resistance, selectively delivering maternal nutrients by the placenta to support fetal growth and development. Insulin sensitivity reduced approximately 50–60% in late pregnancy compared with pre-pregnancy in those with GDM and similarly in those with healthy pregnant women (6, 7). Thus, the primary contributory factor of hyperglycemia is a rise in insulin resistance along with the pancreatic cells’ inability to appropriately react to an increase in insulin release.

The placenta secretes a variety of hormones, such as human placental lactogen (hPL), placental growth hormone (pGH), estrogen, and progesterone, which contribute to preserving pregnancy (8). The increased secretion of hPL and pGH during pregnancy have been shown to cause β-cell proliferation and insulin resistance, respectively. However, changes in placental hormones has no direct correlation with changes in maternal insulin resistance (9). This suggests that mechanisms other than placental hormones may play a key role in mediating the changes in the mother’s metabolism during pregnancy. Exosomes are lipid-based nanovesicles with a bilayer structure that are created in the endosomal pathway and released into biofluid compartments through the fusion of multivesicular bodies with the plasma membrane. Exosomes carry multiple types of signaling molecules, such as lipids, proteins, and RNAs, including microRNAs and mRNAs, which can be delivered to proximal and distal target cells to facilitate intercellular communication.

During pregnancy, placenta-derived exosomes (pdEs) have been detected in maternal circulation as early as 6 weeks of gestation (10). The concentration of both circulating total and placenta-derived exosomes in pregnant women who continue to develop GDM is significantly higher than those in normal healthy pregnancies (11). Moreover, a specific set of miRNAs contained in pdEs isolated from GDM women is associated with skeletal muscle insulin signaling (12). It’s interesting to note that mice that were continuously given small extracellular vesicles (sEVs) enriched from GDM women developed glucose intolerance. Glucose-stimulated insulin secretion (GSIS) and muscle basal insulin signaling were also decreased in mice given GDM sEVs (13). Altogether, these studies suggest that pdEs might play a significant role in regulating maternal glucose metabolism under both normal and pathological conditions during pregnancy.

The state of GDM was quickly alleviated once the placenta was delivered because it is well known that the placenta plays a vital role in causing the insulin resistance found in this condition.However, no studies have explored the molecular mechanisms of pdEs on maternal islets function. Therefore, we investigated whether the human placenta might secrete exosomes carrying specific messages to the pancreas, which contributes to the phenotype of islets dysfunction associated with GDM.

## Materials and methods

### Animal model

Four-week-old male and female C57BL/6 mice were purchased from Shanghai SLAC Laboratory Animal Co., Ltd. The animals were housed at a constant temperature and humidity under a 12-h light-dark cycle. All of the animal procedures were carried out in accordance with the National Institutes of Health guidelines and approved by the animal care committee of Drum Tower Hospital, which is affiliated with Nanjing University Medical School, Nanjing, China. The female mice were fed either a high-fat diet (HFD; 60% of energy from fat from Research Diets, D12492) or a regular diet (standard chow) (10% of energy from fat) from 4 weeks of age throughout the experiment. To induce GDM, HFD female mice were mated overnight at 10 weeks of age and mating was confirmed by the presence of a vaginal plug the following morning, which was marked gestational day (GD) 0.5, continuing the high-fat diet throughout pregnancy. For normal control pregnant (CTR) mice, the normal diet female mice were also mated at the same time, receiving standard chow throughout the experiment. The remain non-pregnant mice were continued fed high-fat or normal diet.

### Intraperitoneal glucose tolerance tests (IPGTTs) and intraperitoneal insulin tolerance tests (IPITTs)

At GD12.5, IPGTTs were conducted in pregnant and nonpregant control-fed and high-fat-fed female mice that were fasted for 16 hours and injected intraperitoneally with 2 g dextrose per kg body weight. For the IPITT, mice received an intraperitoneal injection of 0.5 U insulin per kg body weight after 6h of fasting. Blood glucose was sampled from the tail at 0, 15, 30, 60,90 and 120 min after the glucose or insulin load with a glucometer. In order to assess the effects of time on a high-fat diet in the absence of pregnancy, one set of nonpregnant females aged 10 and 12 weeks was applied as the control for the high-fat-fed nonpregnant females.

### Selection of participants

At the Nanjing Drum Tower Hospital (Nanjing, China), term human placentas (>37 weeks of gestation) were acquired from elective cesarean or natural deliveries of healthy pregnancies and pregnancies with GDM. GDM was identified utilizing a 75 g oral glucose tolerance test (OGTT) at 24–28 weeks, with cut-offs set in consistent with the 2010 International Association of Diabetes and Pregnancy Study Groups criteria [75 g-OGTT fasting blood glucose (FBG) ≥5.1 mmol/L and/or a 1 hour blood glucose (BG) ≥10 mmol/L and/or a 2 hour BG ≥8.5 mmol/L]. Patients with multiple pregnancies as well as patients with pre-existing diabetes mellitus, gestational hypertension, severe hematological, cardiac, psychiatric, renal, hepatic and other immune disease were excluded. Women with normal OGTT were defined as the control group, and their age, gestational age, pregravid body mass index (BMI) and gestational weight gain were matched with that of women with GDM. The present study was approved by the Scientific Research Ethics Committee of the drum tower Hospital (2019-284-01), and informed consent signature was obtained from all of the participants.

### Placental explant culture

Human placental tissues were collected from the villous tree. Within 1 h of delivery, the top 2 mm of the maternal aspect of the placenta was dissect and discard, then three areas of approximately 2cm^3^ of the underlying villous placental tissue were dissected and sampled. For mouse placental explant culture, the animals for preparation of pdEs were chosen randomly. All placentae from one mouse, varying in number from 4 to 11 per mouse, were pooled together to isolate pdEs at GD12.5. Chorionic villi explants were sufficiently washed in PBS and then cut into smaller pieces. Placental explants were cultured in advanced DMEM/F12 medium supplemented with 10% exosome-depleted FBS and 1% penicillin/streptomycin. Chorionic villi were cultured in an incubator set at 37°C and 5% CO2 for 24 hours before exosome isolation.

### Exosome isolation

Exosomes were isolated from culture medium of chorionic villi via differential centrifugation as previously described (14, 15). Briefly, the culture medium was centrifuged at 300g for 5 min, 3000g for 30min, and 10000g for 60 min at 4°C to remove the cellular debris and shedding vesicles. After that, supernatants were filtered through a 0.22μm sterile filter (Millipore) and then centrifuged at 100000g for 70min at 4°C. The exosomes were then resuspended in PBS and preserved for further use at -80°C.

### Primary pancreatic islet isolation and cell culture

8-week-old normal chow male mice are used for islet isolation. Collagenase type V was used to degrade the pancreas, as previously described (16), and then Histopaque density gradient was used to separate the islets from the digested exocrine tissue.After the gradient centrifugation, islets were hand-picked and cultured in RPMI 1640 medium (glucose: 11.1 mmol/L) containing 10% exosome-depleted FBS, 100 IU/mL penicillin, and 100 mg/mL streptomycin at 37°C in a humidified atmosphere consisting of 5% CO2. Then, the islets were counted, replaced into 6- or 48-well plates and cultured overnight for further examination.

The mouse pancreatic b-cell line MIN6 was cultured at 37°C in a humidified 5% CO2 environment in DMEM containing 15% FBS, 100 units/mL penicillin, 100 mg/mL streptomycin, 10 mmol/L HEPES, and 50 mmol/L b-mercaptoethanol.

### *In vivo* and *in vitro* treatment with exosomes

For *in vitro* treatment, placenta-derived exosomes were labeled with a PKH26 Fluorescent Cell Linker Kit (Sigma) according to the manufacturer’s instructions and washed with PBS followed by ultracentrifugation at 100,000×g for 70 min at 4°C. Then, PKH26-labeled exosomes were cocultured with MIN6 cells or islets for 24 hours at a final concentration of 50 ug/ml. The uptake of exosomes was photographed with a confocal fluorescence microscope. For *in vitro* treatment, islets from control C57Bl/6J mice were incubated with pdEVs from CTR and GDM mice at a final concentration of 50 ug/ml, GSIS and TUNEL staining were measured after 24 hours, and apoptosis was determined after 48 hours.

For in vivo treatment, exosomes were labeled with fluorescent lipophilic tracer DiR (Sigma). In brief, DiR was added to an exosomes-PBS mixture at a final concentration of 1 μM and was incubated for 20 min at room temperature in the dark, followed by an additional ultracentrifugation to remove excess dye. Then, labeled exosomes were injected into female C57BL/6J mice via tail vein, and the red fluorescence was detected using an IVIS Spectrum system (Caliper Life Sciences, USA). The organs, which included the brain, heart, lung, liver, spleen, pancreas, and kidney, were removed 6 hours later and quantitated ex situ using the same imaging procedure. For *in vivo* treatment, a single administration of 50 μg pdEVs from CTR and GDM mice were adoptively transferred into normal-diet-fed C57BL/6 pregnant mice at GD12.5 via tail vein injection. In the control groups, PBS was used. On Day 4, IPGTTs were conducted in nomal-diet-fed C57BL/6 pregnant mice.

### Glucose-stimulated insulin secretion (GSIS)

The primary islets were planted in 48-well plates overnight for GSIS assays. After pre-incubated for 1h in HEPES-balanced Krebs-Ringer bicarbonate buffer (KRBH) containing 3.3 mM glucose and 0.2% BSA, the islets were then incubated for 1 h in KRBH with low glucose (3.3 mM) and high glucose (16.7 mM), respectively. Subsequently, the supernatant was removed and kept at −80°C for later determination.

The insulin concentration and insulin content were measured by ELISA, and the insulin secretion index was calculated by the ratio of high glucose to low glucose-stimulated insulin levels.

### Apoptosis

The MIN6 cells or islets were rinsed twice with cold PBS before being resuspended in 1X binding buffer. Dissociated islet cells were obtained by incubating the islets in 0.002% trypsin for 8-15min at 37°C. Then, 5 μl of PE Annexin V and 5 μl 7-AAD were added to MIN6 cells or dissociated islet cells and incubated for 15min at room temperature in the dark. The apoptosis of the cells was detected using flow cytometry (BD, Accuri™C6 Plus).

### TUNEL staining

Terminal deoxytransferase‐mediated dUTP‐biotin nick end labeling (TUNEL) assay was performed to detect the level of apoptosis. According to the manufacturer’s instructions, TUNEL staining was carried out using the One Step TUNEL Apoptosis Assay Kit (C1090, Beyotime). Islets were observed under an inverted fluorescence microscope (Leica, Germany).

### RNA extraction and real-time PCR

Total RNA was extracted by TRIzol according to the manufacturer’s recommendation (Invitrogen, USA). The PrimeScript RT Master Mix (Takara, Cat # RR036A) was used to reverse-transcribe mRNA from 1.0 ug of total RNA. The miRNA 1st Strand cDNA Synthesis Kit (Vazme, China) was utilized for miRNA reverse transcription. Quantitative mRNA/miRNA expression were done using a LightCycler 480 SYBR Green (Roche, Germany) and miRNA Universal SYBR qPCR Master Mix (Vazyme, China) respectively, according to the manufacturer’s guidelines and a LightCycler 480II Real-Time PCR system (Roche, Switzerland), using specific primers. The mRNA levels were normalized to β-actin and U6 small nuclear RNA was employed as an internal control for miRNAs. Analysis of the expression levels was done using the 2Ct comparative method. The primers for mRNA, miRNA, and U6 are presented in **Supplementary Table 1**.

### MiRNA microarray assay

MiRNA microarray was performed by a commercial service (Shanghai OE Biotech Co., Ltd, China). Briefly, total RNAs were extracted from human placental exsomes (n = 6, 3 from GDM and 3 NGT). Total RNA was quantified by the Agilent Bioanalyzer 2100 (Agilent Technologies). The extracted RNA (100ng) was labeled and hybridized to an Agilent Human miRNA Microarray Kit (Release 21.0, 8 × 60K), and this microarray contains 2570 probes for mature miRNA. Signal scanning and analysis were performed using Affymetrix equipment (Affymetrix GeneChip Operating Software). Feature Extraction software (version10.7.1.1, Agilent Technologies) was used to analyze array images to get raw data. Next, Genespring software (version 14.8, Agilent Technologies) was employed to finish the basic analysis with the raw data. To begin with, the raw data was normalized with the quantile algorithm. The probes with all the samples in one group flagged “Detected” were selected for further data analysis. Differentially expressed miRNAs were selected. The threshold for up- and down-regulated miRNAs was fold change ≥ 2.0 and false discovery rate (q value) ≤ 0.05.

### MiRNA mimic transfection

MiRNA duplex mimics and negative controls were obtained from RiboBio (Guangzhou, China). For transient transfection, Lipofectamine 3000 reagent (Thermo) was mixed with miRNA mimics and negative controls as previously described (17). After 48 hr, the transfection efficiencies were validated by qPCR analysis.

### Statistical analysis

At least three independent experiments were performed(At least three preparations of pdEs from different GDM or NGT women or mice, as well as at least three different islet preparations from different animals). Data were processed using GraphPad Prism 5.0 or SPSS Statistics 23 (IBM) software. Comparisons were performed using the Student t test between two groups or ANOVA in multiple groups. Results are presented as Mean ± SEM, p < 0.05 is considered statistically significant.

## Results

### The changes of PdEs and β cell function between normal and GDM pregnancies

**Figure 1:**
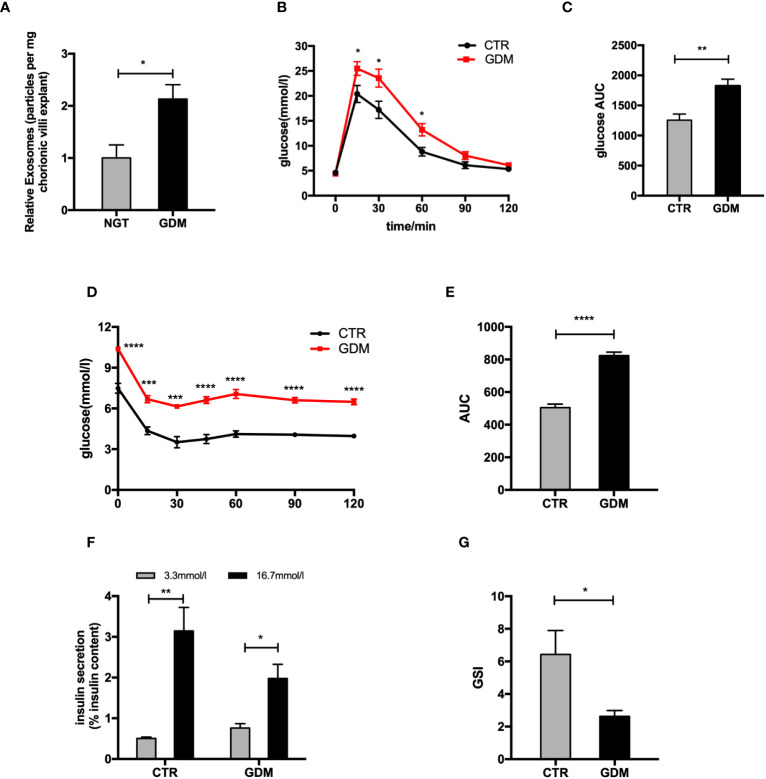
The changes of placenta-derived exosomes and β cell function between normal and GDM pregnancies. **(A)** Concentration of exosomes from normal and GDM pregnancies presented as relative number of particles/mg tissue/24 h. **(B–E)** Glucose tolerance test (GTT) **(B, C)** and insulin tolerance test (ITT) **(D, E)** were performed on a cohort of high-fat (GDM)- and control (CTR)-fed pregnant dams at gestational day 12.5 (GD12.5) (n = 6 per group, CTR and GDM in graph, respectively). **(F, G)** Glucose-stimulated insulin secretion (GSIS) **(F)**, insulin-stimulated index (GSI) **(G)** during GSIS tests. Data are presented as mean ± SEM. *p < 0.05, **P < 0.01, ***p<0.001, ****P < 0.0001, Student’s t test **(A–G)**.

Human and mouse primary placenta were isolated and cultured *in vitro*, and exosomes were purified from the culture conditioned medium by differential centrifugation and ultracentrifugation. The exosomes, which showed a cup-shaped morphology by transmission electron microscopy (TEM) observation, presented diameters of 50-150nm on nanoparticle tracking analysis (NTA) (**Supplementary Figures 1A, B**). Further, western blot analysis confirmed that the particles expressed CD63, ALIX (ALG-2-interacting protein), and tumor susceptibility gene 101 (TSG101) (**Supplementary Figure 1C**). Encouragingly, the concentration of exosomes (exhibited as particles/mg tissue/24 h) from human GDM pregnancies significantly increased during pregnancy compared with NGT (**Figure 1A**), indicating that pdEs might associate with maternal glucose metabolism under pathological conditions during pregnancy.

To explore changes of islets function between GDM and NGT pregnancies, we produced a GDM model. The glucose tolerance testing (GTT) and insulin tolerance testing (ITT) showed that the pregnant mice fed HFD (GDM mice) had impaired glucose tolerance (**Figures 1B, C**) and insulin tolerance (**Figures 1D, E**) compared to control animals at GD 12.5. In addition, the primary islets from GDM mice had significantly decreased glucose-stimulated insulin levels (**Figures 1F, G**) compared to controls. A cohort of nonpregnant HFD- and chow-diet-fed mice did not demonstrate differences among body weight (GD 0.5) and GTT (GD 12.5) (**Supplementary Figures 1D–F**), verifying that this model is truly gestation-dependent and age-matched.

### PdEs from GDM promoted β cell apoptosis and impaired the GSIS *in vitro*

**Figure 2:**
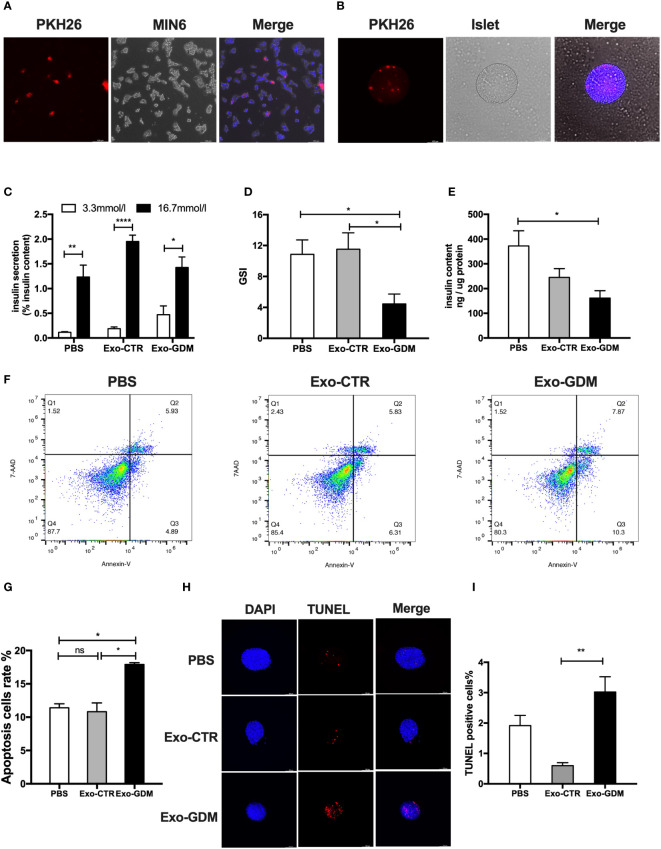
GDM placenta-derived exosomes impaired islets function and survival *in vitro*. Islets of control C57Bl/6J mice were incubated in the presence of PBS, Exo-CTR(pdEs from control pregnant mice), and Exo-GDM(pdEs from GDM mice). Glucose-stimulated insulin secretion (GSIS) and TUNEL staining were measured after 24 hours, and apoptosis was determined after 48 hours. **(A, B)** Mice placenta-derived exosomes uptake in MIN6 cells **(A)** and islets **(B)** after 24 h of co-culture. (Scale bar, 100 μm). **(C–E)** Glucose-stimulated insulin secretion (GSIS) **(C)**, insulin-stimulated index (GSI) **(D)**, insulin content **(E)** during GSIS tests. (n = 8 islets per group, PBS, Exo-CTR, and Exo-GDM in graph, respectively). **(F, G)** Apoptosis was measured by 7AAD-Annexin V test and presented by histogram graph. **(H, I)** Fluorescence microscopy of treated islets stained with TUNEL signals (red) and DAPI (nucleus, blue). (Scale bar, 5μm). TUNEL-positive cells were counted. Data are presented as mean ± SEM. n = 4-7 mice for PBS, Exo-CTR, and Exo-GDM groups.*p < 0.05, **P < 0.01, ****p<0.0001, Student’s t test **(C)**, 1-way ANOVA with Bonferroni’s post- test **(D–I)**. Exo-CTR, placenta-derived exosomes from control pregnant mice, Exo-GDM, placenta-derived exosomes from GDM mice.

To explore the potential influences of pdEs upon islets function and survival, we first tested whether these pdEs can be absorbed by islets cells *in vitro*. Fluorescent dye PKH26 labeled-mice pdEs were put into the culture medium of MIN6 cells or islets. After 24h, red fluorescence was detected in the MIN6 cell or islets, exhibiting efficient uptake of the pdEs (**Figures 2A, B**). Then, islets isolated from normal-diet-fed C57BL/6J mice were treated with PBS, Exo-CTR (pdEs from control pregnant mice), and Exo-GDM (pdEs from GDM mice). Compared to the PBS and Exo-CTR treatments, administration of Exo-GDM significantly decreased GSIS of islets from normal-diet-fed C57BL/6J mice, as evidenced by a lower insulin secretion index (GSI) (**Figures 2C, D**). In addition, Incubation of islets in the presence of Exo-GDM decreased insulin content (**Figure 2E**). Strikingly, cell death of islets treated with Exo-GDM was higher detected by Annexin V or by TUNEL staining (**Figures 2F–I**). Collectively, these data demonstrated that pdEs from GDM mice decreased islets GSIS and increased islets apoptosis.

### PdEs from GDM impaired the glucose tolerance of pregnant mice *in vivo*

**Figure 3:**
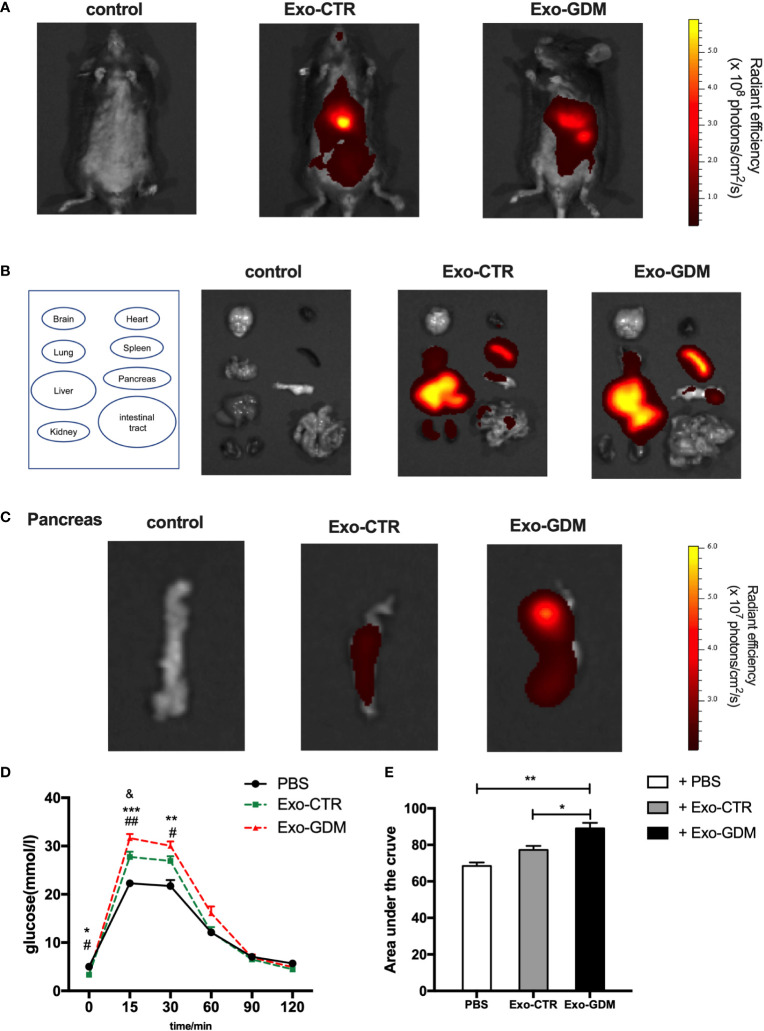
GDM placenta-derived exosomes impaired the glucose tolerance in pregnant mice *in vivo.* PdEs produced by chorionic villi explants isolated from GDM mice and normal pregnant mice were injected via tail vein into normal chow diet pregnant mice, respectively. Red fluorescence was detected using an IVIS Spectrum system. **(A)** *In vivo* fluorescent imaging of mice from each experimental group were taken after 6 h post the pdEs injection. **(B)** Representative IVIS images of seven different organs including the brain, heart, lung, liver, spleen, pancreas, and kidney collected at 6 h following i.v. transfusion of PBS or pdEs, respectively. **(C)** PdEs signal (red fluorescence) in pancreas at 6h post injection. (n = 3 per group). **(D, E)** Glucose tolerance test (GTT) was performed on control pregnant recipient mice after adoptive transfer of PBS, Exo-CTR(placenta-derived exosomes from control pregnant mice), and Exo-GDM(placenta-derived exosomes from GDM mice) at GD16.5. Data are presented as mean ± SEM. *p<0.05, ** p<0.01, *** p<0.001, Exo-GDM vs PBS; # p<0.05, ## p<0.01, Exo-CTR vs PBS, & p<0.05, Exo-CTR vs Exo-GDM, 1-way ANOVA with Bonferroni’s post- test.

Given the marked *in vitro* effects of Exo-GDM, we evaluated their effects on glucose tolerance *in vivo*. To detect the biodistribution of pdEs derived from GDM and CTR pregnant mice *in vivo*, we injected DiR labeled-mice exosomes into mice fed a chow diet via tail vein. *In vivo* fluorescent imaging, which were taken after 6 h post the pdEs injection, demonstrated that DiR fluorescence was mainly lacated in the liver, spleen, and lung of mice (**Figures 3A, B**). Then, we also detected the pdEs signal (red fluorescence) in pancreas after the mice were sacrificed at 6h post injection (**Figure 3C**), suggesting that pdEs can be taken up by different tissues like liver, adipose tissue, pancreas, kidney, and muscle and that HFD consumption could enhance pdEs uptake into pancreas.

Next we evaluated the effects of pdEs on glucose tolerance *in vivo*. Strikingly, glucose tolerance was significantly impaired in normal-diet-fed C57BL/6 pregnant mice injected via tail vein with Exo-GDM (pdEs from GDM mice) as compared to Exo-CTR(pdEs from control pregnant mice) infusion (**Figures 3D, E**). In addition, we also observed decreased glucose intolerance after Exo-GDM treatment compared with PBS control (**Figures 3D, E**). Taken together, pdEs from GDM mice administration can impair glucose intolerance in chow-diet-fed pregnant mice *in vivo*, indicating that placenta might transmit exosomes to pancreas that accentuated islets maladaptation resulting in pregnancy hyperglycemia.

### Expression of candidate miR-320b in placenta-derived exosomes from human NGT and GDM pregnancies

**Figure 4:**
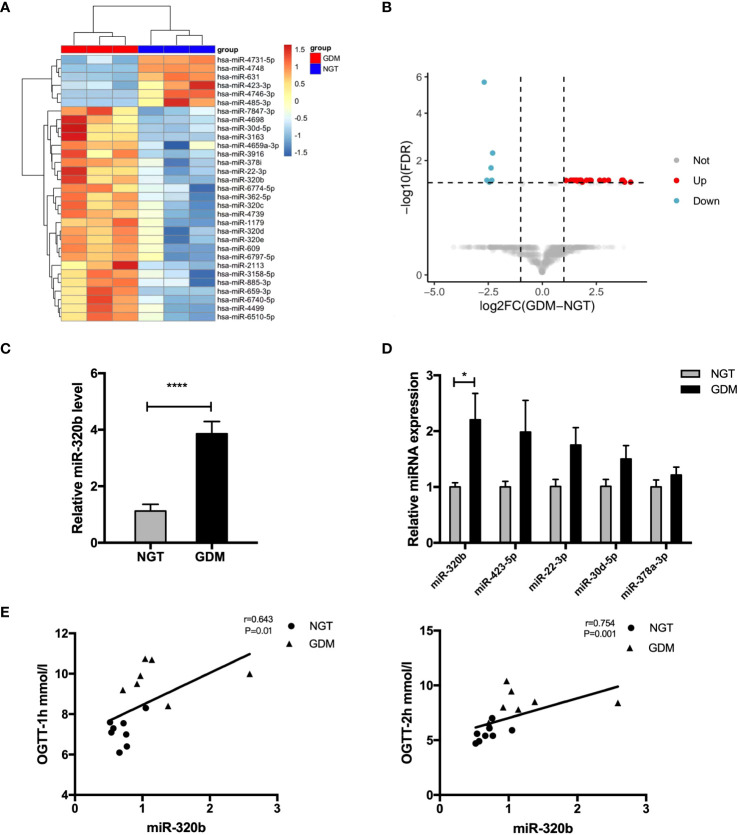
Expression of candidate miR-320b in placenta-derived exosomes from human NGT and GDM pregnancies. **(A, B)** Heatmap **(A)** and volcano plot **(B)** of the upregulated and downregulated placenta exosomal miRNAs in NGT and GDM pdEs (n = 3 for NGT and GDM groups, selectively). MiR-320b is one of the exosomal miRNAs with markably greater abundance in GDM pdEs compared to NGT pdEs. **(C)** Expression level of miR-320b in NGT and GDM pdEs. **(D)** The levels of miR-320b, miR-423-5p, miR-22-3p, miR-30d-5p, miR-378a-3p in placenta from NGT and GDM pregnancies were measured by qPCR analysis. **(E)** The level of miR-320b was analyzed with one-hour blood glucose (BG) and two-hour BG of 75-g OGTT in the second trimester by the Spearman correlation analysis. Data are presented as mean ± SEM. n = 5-8 for NGT and GDM groups. *p<0.05, ****p<0.0001, Student’s t test **(C, D)**.

Studies suggested that exosomes took on important functions on cellular communication through the transportation of miRNAs to convey regulatory signals across various tissues (18, 19). Thus, we determine miRNAs profiles differentially expressed in pdEs from human NGT and GDM pregnancies via a human Aligent miRNA array. The detail demographic information for each group were listed in the **Supplementary Table 2**. A heat map (**Figure 4A**) and a vocano plot (**Figure 4B**) represented that 25 miRNAs were upregulated and 6 miRNAs were downregulated (fold change > 2, q-value<0.05) in GDM pdEs compared with NGT. The up- and down-regulated miRNAs in GDM pdEs are listed in **Supplementary Table 3**.

Among the identified miRNAs, miR-320b reportedly differentially expressed in plasma of prediabetes and newly diagnosed type 2 diabetes (20). It has also been demonstrated that miR-320b suppressed cell proliferation through targeting c-Myc in human cancer cells (21). Our results show that miR-320b was significantly up-regulated in pdEs and placenta tissue of human GDM compared with control pregnancies (**Figures 4C, D**). Strikingly, the level of miR-320b was significantly positively correlated with one-hour BG and two-hour BG of 75-g OGTT taken in 24-28 weeks of human gestation (**Figure 4E**), indicating that miR-320b is associated with the development of abnormal glucose metabolism in GDM. Collectively, these data demonstrated that miR-320b was differentially expressed in pdEs of human GDM and NGT and associated with abnormal glucose metabolism in human pregnancies.

### Placenta-derived exosomal miR-320b promoted islets dysfunction

**Figure 5:**
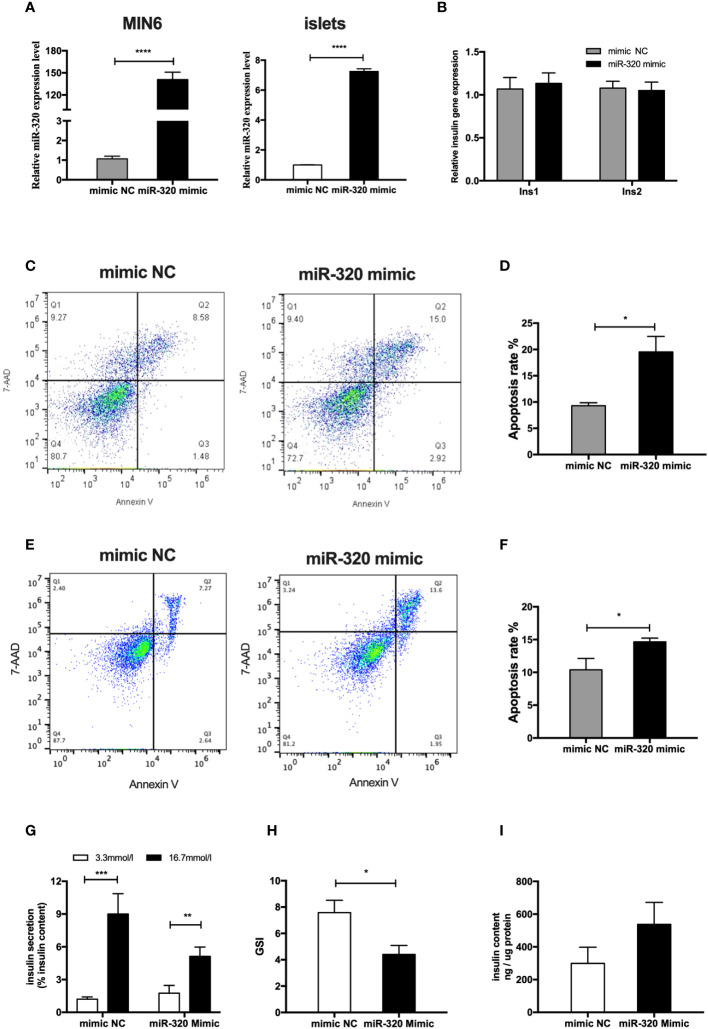
MiR-320b impaired islets function and survival *in vitro*. **(A)** miR-320 mimic and negative control were transfected to MIN6 cells and islets for 48h(n=3 for mimic NC and miR-320 mimic groups, selectively). **(B)** Insulin genesis of MIN6 transfected with miR-320 mimic and negative control was measured by RT-PCR analysis(n=3 per group). **(C–F)** Apoptosis of MIN6 cells **(C, D)** and islets **(E, F)** were assessed by 7AAD-Annexin V test(n=3 per group). **(G-I)** Glucose-stimulated insulin secretion (GSIS) **(G)**, insulin-stimulated index (GSI) **(H)**, insulin content **(I)** during GSIS tests after 48h transfection(n = 8 islets per group, mimic NC and miR-320 mimic in graph, respectively). Data are presented as mean ± SEM. *p < 0.05, **P < 0.01, ***p<0.001, ****p<0.0001, Student’s t test **(A–I)**.

To determine whether the miR-320b mediate the deleterious impact of GDM pdEs on β-cell function and survival, miR-320 mimics were used to significantly increase the miR-320 level in MIN6 cells and islet cells (**Figure 5A**). The results exhibited that miR-320 overexpression obviously induced apoptosis in both MIN6 cells (**Figures 5C, D**) and islet cells after 48h transfection (**Figures 5E, F**). Moreover, overexpression of miR-320 significantly decreased GSIS, as evidenced by a lower GSI (**Figures 5G, H**), whereas miR-320 did not affect the alteration of insulin genesis and insulin content of islets cells (**Figures 5B, I**). In general, these results suggest that miR-320b induced β-cells apoptosis and insulin secretion dysfunction. Based on the evidence above, we supposed that the pdEs might transmit specific miRNAs like miR-320b from human placenta to pancreas that facilitates islets dysfunction phenotype associated with GDM.

## Discussion

Current research suggests that exosomes derived from the placenta participate in maternal changes of islets maladaptation during GDM. Specifically, pdEs from GDM significantly promoted β cell apoptosis and impaired the GSIS *in vitro*. *In vivo* experiments showed that treatment with GDM pdEs directly caused impaired glucose intolerance in pregnant mice.Among the identified differentially expressed miRNAs within pdEs, miR-320b led to increased β cell apoptosis and impaired the GSIS. Thus, exosomes might transfer miR-320b from human placenta to pancreas, which promotes the islets dysfunction phenotype associated with GDM.

Although the mechanisms underlying maternal metabolic adaptation in a healthy pregnancy and metabolic instability in GDM were still unclear, sEVs, especially exosomes, may draw a new picture for regulating maternal glucose homeostasis during pregnancy (22, 23). Studies have shown that the concentration of circulating exosomes (both total and placenta-derived) increases in women with GDM (11). The findings of the study corroborate these findings, with an increase in pdEs isolated from GDM women compared to normal healthy pregnant women. Emerging evidence shows that exosomes play a vital role in modulating maternal physiology in pregnancy. Compared with exosomes from normal pregnancies, exosomes from maternal plasma of GDM accelerate the release of endothelial cytokines (11). Additionally, one study showed that pdEs from GDM women reduced the cell migration and led to insulin resistance in skeletal muscle (12). Furthermore, total sEVs derived from women with healthy pregnancies have been demonstrated to induce pancreatic β-cell GSIS and skeletal muscle insulin resistance in nonpregnant mice. In contrast, sEVs from GDM were unable to promote GSIS and induced aggravated insulin resistance (13). These suggested that sEVs are responsible for the pathophysiology of GDM, containing decreased insulin sensitivity in peripheral tissues and dysregulated endogenous insulin release; however, the specific role of exsomes secreted by the placenta in mediating maternal islets dysfunction developed in GDM is unknown. We focus on the effect of placental exsomes on islets dysfunction developed in GDM. Results from our study showed that pdEs isolated from GDM mice fail to promote GSIS and increased islets apoptosis in islets isolated from mice fed a chow diet. Our findings also demonstrated that compared to treatment with pdEs from control mice, pregnant mice treated with pdEs from GDM mice showed significantly impaired glucose tolerance. It is speculated that the observed effects in our study may be mediated by pdEs.The differences between glucose tolerance in the presence of Exo-CTR and Exo-GDM, although statistically significant, are modest. We hypothesized that this may be due to the inability of injected exosomes to mimic physiological effects and the presence of varying degrees of depletion in the circulation. These results indicate that the altered contents of pdEs have a crucial effect on islets maladaptation, resulting in the changes of maternal metabolism during GDM pregnancies.

To gain further understanding of the molecular mechanisms engaged in the signal transmission of placental exosomes, the miRNA profile of the exosomes in both NGT and GDM women were analyzed. Our results showed that 25 miRNAs were upregulated and 6 miRNAs were downregulated significantly (fold change > 2, q-value<0.05) in GDM pdEs compared with NGT. While analyzing the overexpressed miRNAs in pdEs from GDM, hsa-miR-320b became a interested candidate as it was demonstrated to be differentially expressed in plasma of prediabetes and newly diagnosed type 2 diabetes (20), and inhibited the proliferation of human cancer cells through targeting c-myc (21). In our current studies, interestingly, miR-320b was upregulated in both placental tissues and pdEs of GDM patients, and its level was significantly positively correlated with one-hour BG and two-hour BG of 75-g OGTT taken in 24-28 weeks of human gestation. These results indicated that placenta-derived exosomal miR-320b might influence glucose metabolic process associated with GDM.

The primary determinant of the pathogenesis of GDM is the maternal pancreatic β-cell maladaptation. So far, there are few reports on the interactions between non-coding RNAs (ncRNAs) and β-cell maladaptation in GDM. One study reported that the miR-96 was downregulated both in peripheral blood and placenta tissues of GDM pregnancies, which targets p21- activated kinase 1(PAK1) and promotes the β-cell proliferation and insulin secretion (24). Other findings showed evidence that miR-335-5p inhibited vasohibin-1, ultimately stimulated the transforming growth factor‐β signaling, resulting in the suppressed β-cell GSIS and aggravated insulin resistance in GDM mice (25). Moreover, miR-503 targeted the mTOR pathway to regulate β-cell function in GDM patients (26) and miRNA-221 protected islet dysfunction by targeting PAK1 to regulate apoptosis, proliferation and insulin secretion in pancreatic β cells of GDM (27). Our findings are consistent with the possibility that the candidate miRNA miR-320b induced β cells apoptosis and decreased GSIS *in vitro*. Therefore, we suggested that exosomes containing a specific set of miRNAs like miR-320b released by GDM placental cells may interact with islets cells to regulate the expression of proteins that impinge on cellular process associated with islets survival and function and contributing to the β-cell maladaptation characteristic for GDM.

While still limited, there is evidence to suggest that the crosstalk between placenta and β cell is mediated by ncRNA contained in exosome (12, 13) and enriches the complex pathophysiological mechanisms of GDM. However, due to the possibility of other miRNAs playing critical functional parts in these pdEs, miR-320b is unlikely to be the only reason for the differences in pancreatic dysfunction in mice treated with pdEs. Additionally, multiple miRNAs in pdEs may induce the insulin resistance and islet dysfunction in a collaborative manner. Further work will need to reveal the complete set of pdEs miRNAs that lead to metabolic alterations.

One of the advantages of the present research is that we used placental exosomes from human NGT and GDM pregnancies. Further, we explored the profiles of differently expressed miRNAs in exosomes from these two groups. Our study also has some limitations. First, in this study, the number of chorionic villi explants from women with healthy and GDM pregnancies are limited. Besides, the correlation between placenta-derived exosomal miR-320b and disease severity in a large population needs to be elucidated. Another limitation of this study is that the specific mechanism of placenta-derived exosomal miR-320b mediated the detrimental influences on the function and survival of β cell are lacking. Further validation is needed to explore the exact molecules or pathways regulated by miR-320b *in vitro* and *in vivo*.

In conclusion, this study suggests that the function and survival of β cells are significantly affected by placenta-derived exosomes containing miR-320b, and this regulates metabolic homeostasis in normal and GDM pregnancies. Our results corroborate the possibility that placental exosomes are involved in the pathogenic mechanisms of islets maladaptation in GDM and therefore might be a conceivable early predictor for this disease. Further studies are urgently needed to elucidate the specific molecular mechanisms by which placental exosomes interact with pancreatic β cells or other organs to alter their physiology.

## Data availability statement

The data presented in the study are deposited in the Figshare repository, DOI: 10.6084/m9.figshare.24820662.

## Ethics statement

The studies involving humans were approved by the Scientific Research Ethics Committee of Nanjing Drum Tower Hospital. The studies were conducted in accordance with the local legislation and institutional requirements. The participants provided their written informed consent to participate in this study. The animal study was approved by the animal care committee of Drum Tower Hospital, which is affiliated with Nanjing University Medical School, Nanjing, China. The study was conducted in accordance with the local legislation and institutional requirements.

## Author contributions

YW: Data curation, Formal analysis, Investigation, Methodology, Visualization, Writing – original draft. YY: Data curation, Formal analysis, Investigation, Methodology, Writing – original draft. SS: Data curation, Formal analysis, Writing – review & editing. ZG: Funding acquisition, Methodology, Supervision, Validation, Writing – original draft, Writing – review & editing. DZ: Funding acquisition, Supervision, Validation, Writing – review & editing. YB: Funding acquisition, Methodology, Supervision, Validation, Writing – review & editing.
