# Supplementary material for: Placenta-derived exosomes exacerbate beta cell dysfunction in gestational diabetes mellitus through delivery of miR-320b
Source: Front Endocrinol (Lausanne). 2024 Jan 8;14:1282075. doi: 10.3389/fendo.2023.1282075 (PMC10800463; doi:10.3389/fendo.2023.1282075)
Supplement: Supplementary file 1 [file DataSheet_1.docx]

**Supplementary material**

**Supplementary methods**

**Transmission electron microscopy (TEM)**

The exosomes were obtained by differential centrifugation and ultracentrifugation. Fix the precipitate with 4% glutaraldehyde, vortex for 5 minutes, and fix at room temperature for 30 minutes. Take a drop of sample and drop it onto the copper mesh of the electron microscope, and adsorb for 30 minutes. Dye with 2% uranyl acetate for 10 minutes. Dry at room temperature overnight prior to visualization.

**Nanoparticle tracking analysis (NTA)**

The pdEs concentration and size distribution were measured using Nanoparticle Tracking Analysis (NTA; Nanosight NS500, Malvern Instruments, Malvern, UK).

**Western blotting**

The identity of exosomes was assessed by western blot analysis, evaluating the presence of the surface proteins, Alix、CD63 and TSG101. Protein concentrations were quantitated using a BCA Protein Assay Kit. protein (20 μg) was loaded on to SDS-polyacrylamide gels. After electrophoresis, the proteins were transferred onto PVDF membranes overnight at 4°C. Following transfer, membranes were blocked in either 5% skim milk in Tris-buffered saline with 0.1% Tween80 (TBS-T). Membranes were incubated at 4°C overnight with primary antibodies: Alix(SC-53540, Santa Cruz Biotechnologies), CD63 (Ab-134045, Abcam) and TSG101 (SC-7964, Santa Cruz Biotechnologies). Membranes were incubated with appropriate secondary antibody (Bioworld). Bands were visualized using enhanced chemiluminescence (EcL; EMd Millipore). Band intensities were quantified using ImageJ software (National Institutes of Health).

**Supplemental Table 1 Primer sequences for qRT-PCR (5’-3’).**

| gene | Forward Primer | Reverse Primer |
| --- | --- | --- |
| *hsa-miR-320b* | GCGAAAAGCTGGGTTGAGA | AGTGCAGGGTCCGAGGTATT |
| *hsa-miR-22-3p* | GCGAAGCTGCCAGTTGAAG | AGTGCAGGGTCCGAGGTATT |
| *hsa-miR-378a-3p* | CGCGACTGGACTTGGAGTCA | AGTGCAGGGTCCGAGGTATT |
| *hsa-miR-130b-3p* | GCGCAGTGCAATGATGAAA | AGTGCAGGGTCCGAGGTATT |
| *hsa-miR-423-5p* | GTGAGGGGCAGAGAGCGA | AGTGCAGGGTCCGAGGTATT |
| *hsa-miR-30d-5p* | GCGTGTAAACATCCCCGAC | AGTGCAGGGTCCGAGGTATT |
| *mus-miR-320b* | GCGAAAAGCTGGGTTGAGA | AGTGCAGGGTCCGAGGTATT |
| U6 | CAAATTCGTGAAGCGTT | TGGTGTCGTGGAGTCG |
| *Ins1* | CCCTTAGTGACCAGCTATAATCAGAGA | ACCACAAAGATGCTGTTTGACAA |
| *Ins2* | ATGTCCCGCCGTGAAGTG | GGTAGGCTGGGTAGTGGTG |


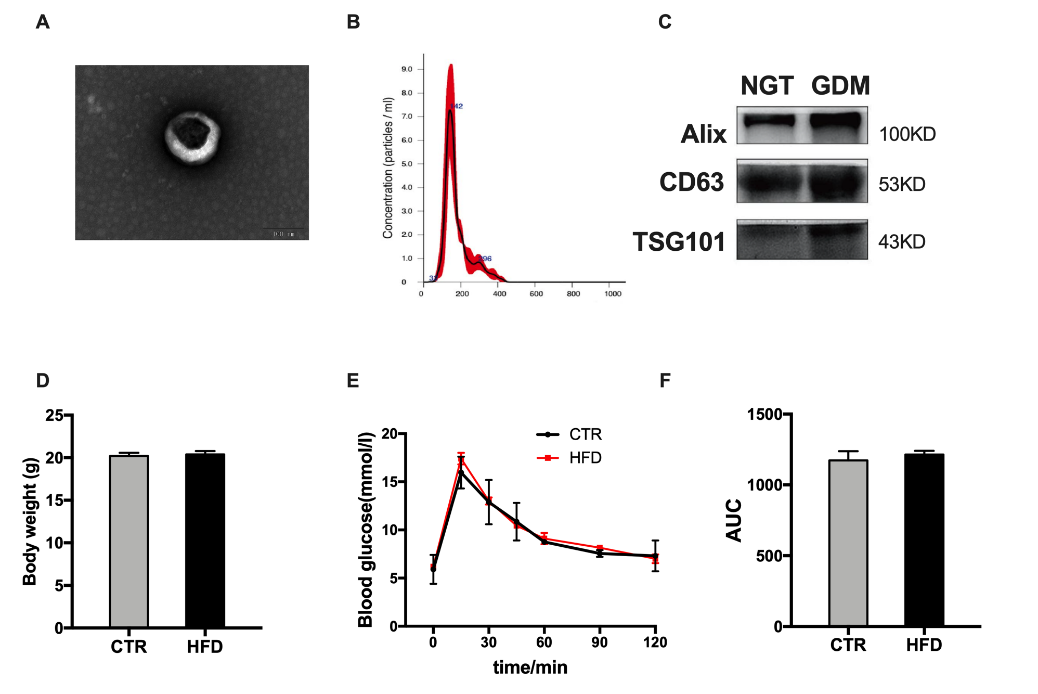


**Supplemental Figure 1. Isolation and Characterization of Exosomes from Chorionic Villi Explants from Pregnancies and glucose tolerance in nonpregnant control-fed and high-fat-fed mice at GD12.5.**

Exosomes were isolated from the culture media of chorionic villi explants from normal and GDM pregnancies by sequential centrifugation.

(A) Visualization of the exosomes released from chorionic villi explants by electron microscopy. Scale bars, 100 nm.

(B) Graphical representation of the vesicle size distribution using a NanoSight technology.

(C) Representative exosome marker Alix, CD63 and TSG101 were measured by western blot analysis. NGT (lines 1) and GDM (lines 2).

(D) The body weight of HFD female mice and control mice at the time of mating. (n = 6 per group).

(E, F) Glucose tolerance test (GTT) (E and F) was performed on a cohort of nonpregnant high-fat (HFD)- and chow-diet (CTR)-fed mice at gestational day 12.5 (GD12.5) (n = 6 per group, HFD and CTR in graph, respectively).

**Supplemental Table 2. Demographic information for GDM and NGT women in miRNA microarray assay.**

|  | NGT | GDM | P |
| --- | --- | --- | --- |
|  | n=3 | n=3 |  |
| Neonatal sex (male/female) | 2/1 | 1/2 | 1.000 |
| Maternal age (year) | 29.0±5.2 | 30.±0.0 | 0.771 |
| Pre-pregnancy weight (kg) | 55.0±3.46 | 55.67±1.15 | 0.768 |
| Maternal height (cm) | 163.67±1.16 | 163.±7.94 | 0.898 |
| Pre-pregnancy BMI (kg/m^2^) | 20.52±0.99 | 21.02±1.58 | 0.669 |
| OGTT at 24-28 gestational weeks | | |  |
| FBG（mmol/L） | 3.96±0.40 | 4.22±0.16 | 0.346 |
| BG-1hour（mmol/L） | 7.23±0.21 | 9.7±1.18 | 0.023 |
| BG-2hour（mmol/L） | 6.23±0.68 | 8.03±0.40 | 0.017 |

**Supplemental Table 3. Up-regulated and down-regulated miRNAs in human GDM pdEs compared with NGT.**

| Up-regulated miRNAs | Down-regulated miRNAs |
| --- | --- |
| hsa-miR-7847-3p | hsa-miR-4731-5p |
| hsa-miR-4698 | hsa-miR-4748 |
| hsa-miR-30d-5p | hsa-miR-631 |
| hsa-miR-3163 | hsa-miR-423-3p |
| hsa-miR-4659a-3p | hsa-miR-4746-3p |
| hsa-miR-3916 | hsa-miR-485-3p |
| hsa-miR-378i |  |
| hsa-miR-22-3p |  |
| hsa-miR-320b |  |
| hsa-miR-6774-5p |  |
| hsa-miR-362-5p |  |
| hsa-miR-320c |  |
| hsa-miR-4739 |  |
| hsa-miR-1179 |  |
| hsa-miR-320d |  |
| hsa-miR-320e |  |
| hsa-miR-609 |  |
| hsa-miR-6797-5p |  |
| hsa-miR-2113 |  |
| hsa-miR-3158-5p |  |
| hsa-miR-885-3p |  |
| hsa-miR-659-3p |  |
| hsa-miR-6740-5p |  |
| hsa-miR-4499 |  |
| hsa-miR-6510-5p |  |
